# Anti-CMV-IgG Positivity of Donors Is Beneficial for alloHSCT Recipients with Respect to the Better Short-Term Immunological Recovery and High Level of CD4+CD25high Lymphocytes

**DOI:** 10.3390/v7031391

**Published:** 2015-03-23

**Authors:** Emilia Jaskula, Dorota Dlubek, Agnieszka Tarnowska, Janusz Lange, Monika Mordak-Domagala, Krzysztof Suchnicki, Mariola Sedzimirska, Agata Borowik, Sylwia Mizia, Andrzej Lange

**Affiliations:** 1 L. Hirszfeld Institute of Immunology and Experimental Therapy, Polish Academy of Sciences, Wroclaw 53-114, Poland; E-Mails: emiljask@wp.pl (E.J.); ddlubek@wp.pl (D.D.); 2 Lower Silesian Center for Cellular Transplantation with National Bone Marrow Donor Registry, Wroclaw 53-439, Poland, E-Mails: agnieszka.tarnowska@poczta.fm (A.T.); jahlange@wp.pl (J.L.); mmordak@o2.pl (M.M.-D.); suchnicki@dctk.wroc.pl (K.S.); sedzimirska@dctk.wroc.pl (M.S.); borowik@dctk.wroc.pl (A.B.); sylwia.mizia@gmail.com (S.M.)

**Keywords:** anti-CMV serostatus, HSCT, lymphocyte reconstitution, CD4+CD25high lymphocytes, GvHD, herpes virus infection

## Abstract

Hematopoietic stem cell transplantation from anti-cytomegalovirus immunoglobulin G (anti-CMV-IgG) positive donors facilitated immunological recovery post-transplant, which may indicate that chronic CMV infection has an effect on the immune system. This can be seen in the recipients after reconstitution with donor lymphocytes. We evaluated the composition of lymphocytes at hematologic recovery in 99 patients with hematologic malignancies post hematopoietic stem cell transplantation (HSCT). Anti-CMV-IgG seropositivity of the donor was associated with higher proportions of CD4+ (227.963 ± 304.858 × 10^6^ *vs.* 102.050 ± 17.247 × 10^6^ cells/L, *p* = 0.009) and CD4+CD25high (3.456 ± 0.436 × 10^6^ *vs.* 1.589 ± 0.218 × 10^6^ cells/L, *p* = 0.003) lymphocytes in the blood at hematologic recovery. The latter parameter exerted a diverse influence on the risk of acute graft-*versus*-host disease (GvHD) if low (1.483 ± 0.360 × 10^6^ *vs.* 3.778 ± 0.484 × 10^6^ cells/L, *p* < 0.001) and *de novo* chronic GvHD (cGvHD) if high (3.778 ± 0.780 × 10^6^ *vs.* 2.042 ± 0.261 × 10^6^ cells/L, *p* = 0.041). Higher values of CD4+ lymphocytes in patients who received transplants from anti-CMV-IgG-positive donors translated into a reduced demand for IgG support (23/63 *vs.* 19/33, *p* = 0.048), and these patients also exhibited reduced susceptibility to cytomegalovirus (CMV), Epstein–Barr virus (EBV) and/or human herpes 6 virus (HHV6) infection/reactivation (12/50 *vs.* 21/47, *p* = 0.032). Finally, high levels (≥0.4%) of CD4+CD25high lymphocytes were significantly associated with better post-transplant survival (56% *vs.* 38%, four-year survival, *p* = 0.040). Donors who experience CMV infection/reactivation provide the recipients with lymphocytes, which readily reinforce the recovery of the transplanted patients’ immune system.

## 1. Introduction

Alloreactivity and poor immune system recovery leading to life-threatening complications post-hematopoietic stem cell transplantation (HSCT) remains a significant challenge for researchers. At present, we believe that the recognition of foreign human leukocyte antigens (HLAs) evokes a vigorous response involving cytokine production, known as a cytokine storm [1,2,3,4]. Although the advanced stage of acute graft-*versus*-host disease (aGvHD) is associated with a level of HLA disparity, a sizeable proportion of patients may still survive despite relatively poor matching [5,6]. Under these conditions, it is believed that T regulatory cells (Tregs) present in the blood can control the vigorousness of the alloreactive response [7,8,9]. Twenty years ago, the phenotype of this cell population was described as CD4+CD25high by Sakaguchi *et al.* [10]. This population meets the criteria for cells that control the immune response using suppressor cell machinery, which functions in cells with interleukin 2 (IL-2) containing environments [11,12,13]. In particular, IL-2-activated CD4+CD25high lymphocytes (forkhead box P3 (FoxP3)+) may exert both specific and nonspecific suppression of the immune response as bystander cells [14,15,16,17]. aGvHD is recognized as a failure of these IL-2-activated CD4+CD25high lymphocytes [18,19]. Notably, seropositivity of donors plays a positive role making recipients less susceptible to aGvHD [20,21,22,23,24]. It is also known that cytomegalovirus (CMV) infection sustained in affected individuals life-long influences the immune system changing the profile of T cells in blood [25]. The fate of HSCT largely depends on the lymphocyte composition of the transplant material [26,27], which is different in patients having and lacking chronic CMV infection [28]. However, the issue on the effect of the donors anti-cytomegalovirus immunoglobulin G (anti-CMV-IgG) seropositivity on aGvHD and survival is controversial. This might be due to the presence of several confounding factors that may bias the final results, including site specific classification of transplant related morbidities in multicenter studies [29,30]. The conclusion of the study by Ljungman *et al.* [31,32,33] suggest that the beneficial effect of anti-CMV-IgG positivity is mediated by donors T cells. Therefore, we focused on the effect of donors IgG CMV seropositivity on the immune system recovery in patients post HSCT.

The novel aspect of our paper is that donor anti-CMV-IgG positivity was associated with a higher proportion of CD4+CD25high lymphocytes, which likely causes the recipient to be less susceptible to aGvHD. In addition, recipients of anti-CMV-IgG negative donors were doing poorly post HSCT in terms of the overall number of CD4+ cells, and they demanded more frequent intravenous IgG support. Finally, patients who presented with a CD4+CD25high lymphocyte proportion ≥0.4% enjoyed better survival than those with the proportions below 0.4%.

## 2. Materials and Methods

### 2.1. Patient Characteristics

In total, 99 patients underwent transplantation at our institution from 2007–2013, and these patients were followed post-HSCT. They received either marrow (BM-4 patients) or peripheral blood progenitor cells (PBPC-94 patients, one patient received PBPC + BM) from matched sibling (SIB: 40 patients) or unrelated donors (MUD: 59 patients). 

All donors were clinically screened according to the WMDA guidelines what including in addition to the routine viral make up and also serological profile of antibodies against herpes viruses (CMV, Epstein–Barr virus (EBV), (herpes simplex virus HSV). A total number of anti-CMV-IgG positive donors equaled 64 individuals. Positive and negative anti-CMV-IgG donors differed with respect to the age (mean ± SEM: 39.2 ± 1.5 *vs.* 32.5 ± 2.2 years old, *p* = 0.014, respectively).

In total, 67 and 32 patients followed myeloablative (MAC) and reduced (RIC) conditioning regimens, respectively. All MUD patients except one also received anti-lymphocyte antibodies: 51 patients received anti-thymocyte antibodies (ATG; Fresenius, Munich, Germany), and 7 patients received alemtuzumab (Campath; Genzyme, Cambridge, MA, USA). Within the SIB group, as part of their conditioning regimen, 13 patients received ATG, and 3 patients received Campath. The patient characteristics are presented in Table 1. All patients received cyclosporine A at a dose adjusted to a trough value of 200 ng/mL. The dose was tapered after three months, and it was usually discontinued by 6 months post-HSCT. Patients with a serum IgG level below 500 mg % received routine intravenous immunoglobulin support (KIOVIG; Baxter, Lessines, Belgium).

The patients were discharged from the hospital between 30 and 60 days post-transplant and were followed on an outpatient basis. The patients were invited to appear at two-week intervals or at any time when symptoms suggesting the presence of post-transplant complications were noticed. Blood work included peripheral blood lymphocyte profiling and microbial/viral surveillance.

The starting point of our lymphocyte study was the first day of hematologic recovery (granulocytes >500/µL; from +6 to +39 days post-HSCT, median: 14 days). At the time of hematologic recovery, 92% patients had chimerism in the blood that exceeded 90%.

aGvHD and cGvHD were diagnosed according to the European Society for Blood and Marrow Transplantation and the National Institutes of Health guidelines, respectively.

Epstein-Barr virus (EBV), CMV, and human herpes virus 6 (HHV6) DNA copies were detected in the blood (from hematologic reconstitution performed 3 times at one-week intervals during the first month post-HSCT and then at one-month intervals until 100 days post-HSCT, followed by 4 times per year during routine check-ups). Viral DNA copy numbers were always assessed when post-HSCT complications were found.

**Table 1:** Patient and donor characteristics.

**Patients**	
Number (*n*)	99
Age (median, range)	45, 6–65
Gender (female/male)	54/45
Type of transplantation (alternative/SIB)	59/40
Transplant material (PBPCs/BM)	94/4 1 PBPCs + BM
**Diagnosis**	
AML	53
ALL	23
Other lymphoproliferative disorders	3
Myeloproliferative disorders	9
Myelodysplastic syndrome	11
Conditioning regimen:	
Myeloablative	67
Reduced intensity	32
**aGvHD grade**	
*0*	55
*I*	14
*II*	13
*III*	5
*IV*	12
**CMV IgG serostatus**	
anti-CMV-IgG negative	20
anti-CMV-IgG positive	78
Data not available	1
**Donors**	
Age (median, range)	37, 19–62
SIB donors (median, range)	44, 19–62
MUD donors (median, range)	31, 20–54
Gender (female/male)	31, 48/51
**CMV IgG serostatus**	
anti-CMV-IgG negative	33
anti-CMV-IgG positive	64
Data not available	2

All patients with herpes virus reactivation except one had >100 DNA copies of CMV, HHV6, and/or EBV per 10^5^ cells in the blood from 1–33 weeks (median: 6 weeks) after CD4+CD25high lymphocyte assessment. Herpes virus infection/reactivation events, including CMV, EBV, and/or HHV6 infection/reactivation, during the first year post-transplant were observed in 48/99 patients.

All donors and recipients gave written consent to the use of their blood work results in scientific elaborations presented on a group basis in an anonymous fashion. The whole project was granted approval by the local Bioethics Committee (KB-52/2010).

The whole group of patients and its subgroups—anti-CMV-IgG donors positive and negative graft recipients, and those with *in vivo* T cells depletion—were tested separately.

### 2.2. Phenotypic Analysis

Whole blood was used for membrane staining and one-step density gradient centrifugation (Lymphoprep: *d* = 1.077 g/mL; Nycomed Pharma AS, Oslo, Norway) was used for intracellular FoxP3 labeling in isolated mononuclear cell fractions. Flow cytometry was performed using a FACSCalibur flow cytometer (Becton Dickinson, San Jose, CA, USA) with CellQuest Pro software (Becton Dickinson) to acquire cells. The following monoclonal antibodies (Mo-Abs) were used for cell surface and intracellular staining after permeabilization: PerCP-anti-CD4, FITC-conjugated anti-CD25, APC-anti-CD45, and PE-conjugated anti-FOXP3 (eBioscience, San Diego, CA, USA).

Phenotypic analyses were performed using WinMDI software. Generally, 20,000 cells were acquired. Lymphocytes were analyzed in the gated population, which lacked cells with granulocyte or monocyte characteristics (CD45high, SSC low). In 16 patients, the proportion of CD4+CD25high lymphocytes correlated with the proportion of FoxP3 + CD4+ lymphocytes (*R* = 0.551, *p* = 0.028).

### 2.3. EBV, CMV, and HHV6 Quantification

The numbers of CMV, EBV, and HHV6 DNA copies in peripheral blood cells were determined using real-time polymerase chain reaction (PCR) and a LightCycler II (Roche, Mannheim, Germany). The sequences of the PCR primers and the probe were selected from the *BALF5* region of EBV, the *US17* region of CMV, and the *U67* region of HHV6. PCR was performed as previously described [34].

### 2.4. Anti-CMV IgG Antibody Quantification

Anti-CMV IgG antibodies were detected in sera with the use of CMV IgG ELISA Test System (Zeus Scientific, Inc. Branchburg, NJ, USA). Briefly, recipient’ and donor’ sera and control samples were incubated in microtitere plate wells pre-coated with inactivated CMV antigen. Specific anti-CMV-IgG antibodies if attached to the coated wells were identified with peroxidase-labeled anti-Human IgG antibodies and the reaction was visualized adding 3',5,5'-tetramethylbenzidine (TMB)—peroxidase substrate. The staining intensity generated after hydrolysis of the peroxidase substrate was read in the spectrometer. The results were interpreted according to the manufacturer’s instructions [22,35].

### 2.5. CRP Quantification

C-reactive protein (CRP) was measured routinely in blood at least twice weekly employing specific antibodies (CardioPhase hsCRP—Simens, Marburg, Germany) and nephelometer readings (BN ProSpec System—Simens, Marburg, Germany).

### 2.6. Statistical Analysis

Statistical analysis was performed using CSS Statistica for Windows (version 10) software (Stat-Soft Inc., Tulsa, OK, USA, 2010). The Mann-Whitney U-test and the Kruskal-Wallis test were used for nonparametric, unpaired samples, and the Kaplan-Meier and log-rank tests were used for overall survival analysis and logistic regression in a multivariate analysis to assess the differences between the investigated groups. Additionally, correlations were calculated using Spearman’s rank correlation test. Differences between samples were considered significant at *p* < 0.05.

## 3. Results

Donor anti-CMV-IgG negativity resulted in (I) lower numbers of CD4+ lymphocytes in the blood at hematologic recovery (102.050 ± 17.247 *vs.* 227.963 ± 304.858 × 10^6^ cells/L, *p* = 0.009); (II) a greater proportion of patients that were on intravenous IgG support during the first 100 days post-HSCT (19/33 *vs.* 23/63, *p* = 0.048); and (III) a higher risk of herpes virus reactivation (21/47 *vs.* 12/50, *p* = 0.032) and that of aGvHD (20/33 *vs.* 23/64, *p* = 0.020). 

**Figure 1:**
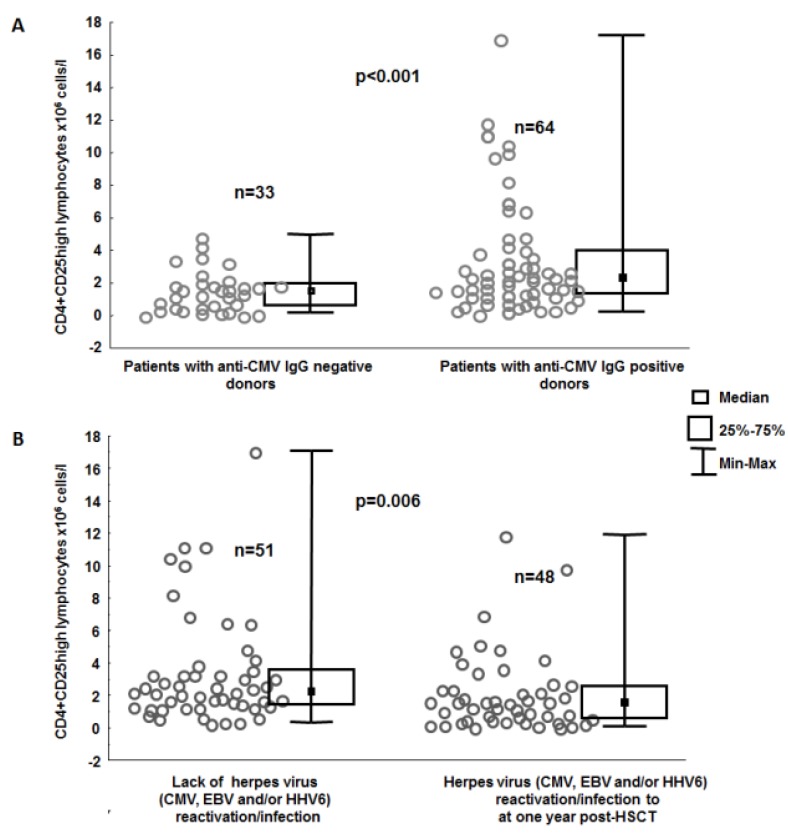
Numbers of CD4+CD25high lymphocytes in the blood determined at the beginning of hematologic recovery in groups of patients stratified according (**a**) to their anti-CMV IgG donor serostatus (**b**) and with or without herpes virus (cytomegalovirus (CMV), Epstein–Barr virus (EBV) and/or human herpes 6 virus (HHV6)) reactivation/infection at one year post-HSCT.

Blood work revealed that the above group of patients receiving a graft from anti-CMV-IgG negative donors had lower proportions and numbers of CD4+CD25high lymphocytes (0.374% ± 0.030% *vs.* 0.503% ± 0.041%, *p* = 0.036; 1.589 ± 0.218 × 10^6^ *vs.* 3.456 ± 0.436 × 10^6^ cells/L, *p* = 0.003, Figure 1A) as compared to their counterparts grafted from CMV IgG positive donors.

Proportions and counts of CD8+ and CD16+CD56+ lymphocytes in the blood at hematologic recovery were investigated against donor anti-CMV-IgG CMV serostatus. It was found that the proportions (29.512% ± 1.741% *vs.* 22.514% ± 2.019% *p* = 0.041) and at the trend level the numbers of CD8+ lymphocytes (240.867 ± 34.251 × 10^6^ cells/L *vs.* 96.850 ± 13.001 × 10^6^ cells/L *p* = 0.062) were higher in patients receiving grafts from anti-CMV-IgG seropositive donors as compared to those grafted from anti-CMV-IgG negative donors, but not when CD16+CD56+ lymphocytes were considered.

Interestingly, an increased risk of herpes virus reactivation (CMV, EBV, and/or HHV6) was associated with reduced levels of CD4+CD25high lymphocytes in the blood (2.180 ± 0.349 × 10^6^ cells/L *vs.* 3.482 ± 0.477 × 10^6^ cells/L, *p* = 0.006; 0.370% ± 0.030% *vs.* 0.539% ± 0.045%, *p* < 0.001; Figure 1B). This association between herpes viruses (CMV, EBV, and/or HHV6 reactivation) and reduced levels of CD4+CD25high lymphocytes was found in the whole-group analysis and in the subgroup of patients who received ATG or Campath (0.320% ± 0.029% *vs.* 0.548% ± 0.072%, *p* < 0.001; 1.445 ± 0.217 × 10^6^ cells/L *vs.* 2.322 ± 0.326 × 10^6^ cells/L, *p* = 0.011). 

When patients receiving grafts from anti-CMV-IgG positive and negative donors were analyzed separately, it appeared that the observation drawn from the whole group analysis was valid for the subgroup composed of patients grafted from anti-CMV-IgG positive donors (2.639 ± 0.582 × 10^6^ cells/L *vs.* 4.015 ± 0.606 × 10^6^ cells/L, *p* = 0.027; 0.414% ± 0.050% *vs.* 0.563% ± 0.059%, *p* = 0.053), but in the anti-CMV-IgG negative donors subgroup reduced proportions (0.320% ± 0.03% *vs.* 0.470% ± 0.050% *p* = 0.022 but not counts of CD4+CD25high (1.467 ± 0.266 × 10^6^ cells/L *vs.* 1.803% ± 0.380 × 10^6^ cells/L, *p* = 0.385) were associated with the risk of herpes virus reactivation.

Multivariate analysis demonstrated that, in terms of the risk of herpes virus (CMV, EBV, and/or HHV6) reactivation (Table 2), the percentage of CD4+CD25high lymphocytes (OR = 0.089, *p* = 0.022), but not that of CD4+ lymphocytes (which failed to reach a significant position in the preliminary steps of the forward step-wise regression analysis), was a significant and independent factor along with CMV serostatus (OR = 4.737, *p* = 0.010) and the level of donor-recipient matching (OR = 3.150, *p* = 0.051). In the subgroup of patients who received ATG or Campath, low percentages of CD4+CD25high lymphocytes (OR = 0.062, *p* = 0.033) and the CMV donor/recipient serostatus (R+/D−) (OR = 5.784, *p* = 0.010) were found to have significant effects (Table 2).

**Table 2:** Multivariate analysis of factors associated with herpes virus (CMV, EBV, and/or HHV6) infection/reactivation.

Parameters	Percentage of CD4+CD25high lymphocytes	HLA mismatch	Anti-CMV IgG serostatus (D−/R+)
**Entire group, *n* = 99**			
**Coefficient**	−2.421	1.147	1.555
***p* value**	0.022	0.051	0.010
**Odds ratio**	0.089	3.150	4.737
**−95% Cl**	−4.489	−0.004	0.389
**+95% Cl**	−0.353	2.298	2.722	
***In vivo* T cell-depleted group (patients receiving ATG or Campath), *n* = 73**			
**Coefficient**	−2.779	1.196	1.755
***p* value**	0.033	0.061	0.010
**Odds ratio**	0.062	3.306	5.784
**−95% Cl**	0.005	0.945	1.537
**+95% Cl**	0.794	11.570	21.764

* Table 2 shows the results of the forward stepwise logistic regression analysis. During the first step analysis, we included factors that had already been suggested [23,34,36,37,38,39] to contribute to the risk of herpes virus reactivation, including the type of donor (sibling or unrelated), the level of donor-recipient HLA matching (9/10 or less *vs.* 10/10 and sibling-matched transplantations), anti-CMV IgG serology (donor serology and donor/recipient serology: negative/positive), mode of transplantation (MAC *vs.* RIC), transplant material (peripheral blood progenitor cells (PBPCs) *vs.* bone marrow (BM)), and the percentages and numbers of CD4+ and CD4+CD25high lymphocytes.

aGvHD was observed in 44 patients, and 14, 13, 5, and 12 of these patients suffered from grades I, II, III, and IV, respectively. A total of 24 of these patients progressed to cGvHD, and cGvHD was diagnosed *de novo* in 21 patients*.* A total of 18 patients died within 100 days of HSCT, and these individuals were excluded from the analysis of cGvHD risk factors.

**Figure 2:**
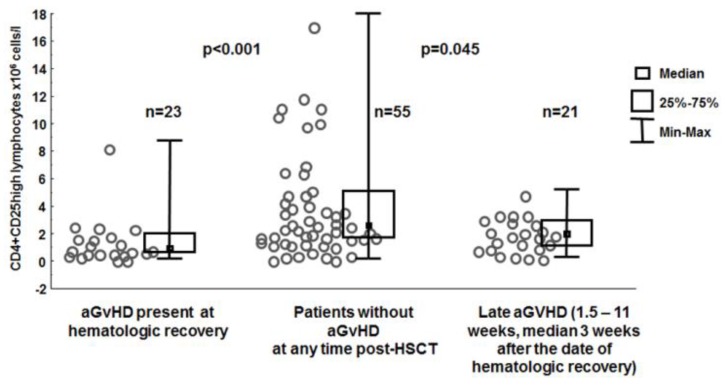
Numbers of CD4+CD25high lymphocytes in the blood at hematologic recovery in patients with and without acute graft-*versus*-host disease (aGvHD) at the time of examination or at a later time point post-transplant.

To analyze in some depth the association between GvHD and proportions/numbers of CD4+CD25high cells in the blood the entire group of patients was subdivided into three subgroups according to the manifestation of aGvHD: (I) absent; (II) appearing at hematologic recovery; or (III) appearing at a later time point post-transplant. The numbers of CD4+CD25high lymphocytes in the blood at hematologic recovery were higher in patients who did not develop aGvHD during the post-transplantation period than in patients who did develop aGvHD during that time (3.778 ± 0.484 × 10^6^ cells/L *vs.* 1.483 ± 0.360 × 10^6^ cells/L, *p* < 0.001), as well as those who developed aGvHD at a later time (10 days to 11 weeks, median: 3 weeks, 3.778 ± 0.484 × 10^6^ cells/L *vs.* 1.921 ± 0.285 × 10^6^ cells/L, *p* = 0.045, Figure 2). Very similar associations were seen in the patients grafted from anti-CMV-IgG positive donors (4.257 ± 0.615 × 10^6^ cells/L *vs.* 2.027 ± 0.375 × 10^6^ cells/L, *p* = 0.012; 0.546% ± 0.365% *vs.* 0.425% ± 0.092% *p* = 0.004;) but not from CMV IgG negative donors (2.152 ± 0.432 × 10^6^ cells/L *vs.* 1.233 ± 0.194 × 10^6^ cells/L *p* = 0.111; 0.317% ± 0.036% *vs.* 0.412% ± 0.043%, *p* = 0. 298).

To validate the hypothesis regarding the effect of toxicity and major infectious complications prior to hematological recovery on the numbers of CD4+CD25high lymphocytes, we examined the toxicity grades and C-reactive protein (CRP) levels in the patient group. Higher CRP values three to five days prior to hematologic recovery were somewhat associated with higher proportions of CD4+ lymphocytes (*R* = 0.267, *p* = 0.008) but not with CD4+CD25high lymphocytes (*R* = 0.018, *p* = 0.858) in the blood. No significant associations between toxicity grades or the proportions or numbers of either CD4+ or CD4+CD25high lymphocytes were observed. 

Additionally, the effect of the use of anti-lymphocyte serum (74% of patients received ATG or Campath) on the association between aGvHD and low numbers of CD4+CD25high lymphocytes was excluded because patients in the *in vivo* T cell-depleted group had lower numbers of CD4+CD25high lymphocytes if they suffered from aGvHD (2.052 ± 0.250 × 10^6^ cells/L *vs.* 5.092 ± 0.776 × 10^6^ cells/L, *p* < 0.001); this finding was also observed in the entire group analysis. A similar finding was also observed in the multivariate analysis, indicating that low numbers of CD4+CD25high lymphocytes (OR = 0.589, *p* = 0.031) and an unrelated transplantation (OR = 2.619, *p* = 0.047) setting were independent and significant risk factors of aGvHD in patients who received and lacked *in vivo* T cell depletion (Table 3).

**Table 3:** Multivariate analyses of the factors associated with aGvHD (grades I–IV).

**Entire group, *n* = 99**				
**Parameters**	**Number of CD4+ lymphocytes (×10^3^ cells/L)**	**Percentage of CD4+CD25high lymphocytes**	**Number of CD4+CD25high lymphocytes (×10^3^ cells/L)**	**MUD donor**
**Coefficient**	0.001	1.394	−0.529	0.963
***p* value**	0.496	0.223	0.031	0.047
**Odds ratio**	1.001	4.031	0.589	2.619
**−95% Cl**	−0.003	−0.863	−1.009	0.014
**+95% Cl**	0.005	3.651	−0.049	1.911
***In vivo* T cell-depleted group (patients receiving ATG or Campath), *n* = 73**				
**Parameters**	**HLA mismatch**	**Anti-CMV IgG serostatus (D−/R+)**	**Number of CD4+CD25high lymphocytes (×10^3^ cells/L)**	**MUD donor**
**Coefficient**	0.600	−0.423	−0.363	1.970
***p* value**	0.347	0.487	0.048	0.029
**Odds ratio**	1.822	0.655	0.696	7.173
**−95% Cl**	0.515	0.196	0.485	1.224
**+95% Cl**	6.450	2.194	0.997	42.016

* Table 3 shows the results of the forward stepwise logistic regression analysis. During the first step analysis, we included factors that had already been suggested [21,23,24,36,40,41,42] to contribute to the risk of aGvHD, including the type of donor (sibling or unrelated), the level of donor-recipient HLA matching (9/10 or less *vs.* 10/10 and sibling-matched transplantations), CMV reactivation events during the first year post-HSCT, age, anti-CMV-IgG serology (donor serology and donor/recipient serology: negative/positive), mode of transplantation (MAC *vs.* RIC), transplant material (peripheral blood progenitor cells (PBPCs) *vs.* bone marrow (BM)), the female-to-male transplantation, percentage and the numbers of CD4+ and CD4+CD25high lymphocytes.

**Figure 3:**
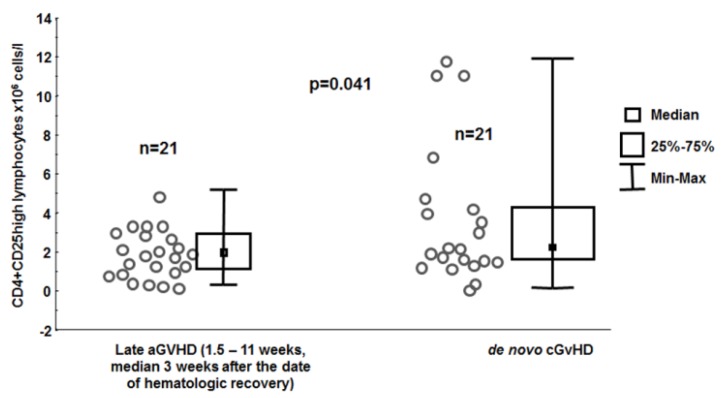
Numbers of CD4+CD25high lymphocytes in the blood determined at the beginning of hematologic recovery in the groups of patients with late aGvHD and those with *de novo* cGvHD.

The CD4+CD25high lymphocyte count at hematologic recovery was able to discriminate patients at risk for acute (low value) or *de novo* chronic (high value) GvHD (3.778 ± 0.780 × 10^6^ cells/l *vs.* 2.042 ± 0.261 × 10^6^ cells/L, *p* = 0.041, Figure 3).

**Figure 4:**
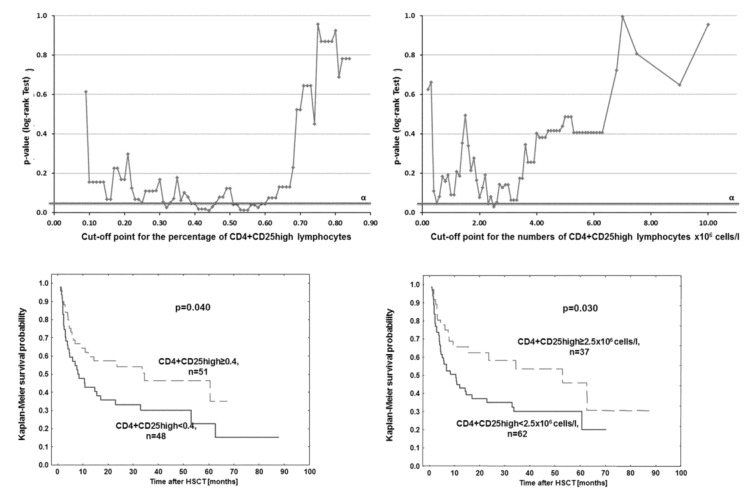
Overall survival of patients with higher and lower proportions and numbers (<0.4% and <2.5 × 106 cells/L, or the discriminative values for proportions and numbers, respectively) of CD4+CD25high lymphocytes (**lower panel**) based on the cut-off point analysis (**upper panel**). The optimal cut-off point selected was the point with the maximum log-rank statistic close to a 50/50 split of patients.

CD4+CD25high lymphocyte levels at hematologic recovery were analyzed in a step-wise manner to determine the values that discriminated patients according to their survival post-HSCT. It appeared that lower proportions (≤0.4%) and numbers (≤2.5 × 10^6^ cells/L) of CD4+CD25high lymphocytes were associated with worse patient survival (56% *vs.* 38%, four-year survival, *p* = 0.040 Figure 4).

## 4. Discussion

CMV infection exerts a profound effect upon the immune system [28]. Several studies performed on mice [43] as well as on people [25] showed that in consequence of CMV infection there is a shift in blood lymphocyte composition in favor of more mature cells. At the beginning of the repetitive CMV reactivation, in young individuals, the potential to combat infection may increase [44,45], but with time the population of mature T cells increases including, CD57+ lymphocytes and those exhausted being PD-1 positive. The latter cells promote Treg cells expansion and functioning. More recently Terrazzini *et al.* [46] documented the fact that CMV-specific iTregs recognizing the same antigens as conventional CD4+ T cells are significantly more frequent in anti-CMV-IgG positive healthy individuals as compared to seronegative ones. They suppressed antigen-specific and nonspecific proliferation and in a large part expressed Foxp3. HSCT is a procedure in which transplanted cells repopulate the patient’s immune system and the transplanted lymphocytes off-spring seen at hematologic recovery in the blood reflect the profile of transplanted lymphocytes in other words reflect the immune system characteristics of the donor. With this knowledge we started research on the profiling of blood lymphocytes at hematologic recovery focusing on CD4+ lymphocytes and its CD25high subpopulation to find out whether anti-CMV-IgG seropositivity of donors influences the repopulation potential of transplanted lymphocytes. The results of this study are in line with previous reports of the lower susceptibility to acute GvHD of patients with higher counts of CD4+CD25high lymphocytes in the blood [47,48,49], but it represents the first documentation of an association between higher levels of CD4+CD25high lymphocytes in the blood and anti-CMV-IgG positivity in transplant donors. The rationale behind these findings can be drawn from several research reports, as quoted below.

CD4+CD25high lymphocytes are either FoxP3 positive (over 80% [50,51]) or promptly express this transcription factor (2–4 h) following IL-2 exposure [52,53] in soluble form or bound to dendritic cells and the extracellular matrix [54]. IL-2 is crucial for the maintenance of regulatory Tregs and for the differentiation of CD4+ T cells into defined effector T cell subsets following antigen-mediated activation, thus favoring either an immune response or suppression. Therefore, counting CD4+CD25high lymphocytes may provide information on the total number of cells equipped with FoxP3 and those ready to express this factor under short IL-2 stimulation. 

Anti-CMV-IgG positivity is closely correlated with the presence of a cellular response in the same individual and reflects primary CMV infection and the presence of the virus in a latent form [55,56]. Reactivation events boost the immune response, promoting the effectiveness of specific surveillance [57].

Reactivation events are usually frequent in anti-CMV-positive healthy donors and result in (I) a skewing of the lymphocyte profile into one that is characteristic of more mature cells, including CD57 positive, CD28 negative and CD26 negative [58,59] lymphocytes that are less prone to active proliferation and (II) the appearance of blood Tregs that are primarily CMV specific and are then able to suppress antigen-specific and nonspecific proliferation [46]. The immediate outcome of HSCT, particularly aGvHD, depends largely on the composition and functional properties of T cells present in the inoculum [60,61,62,63]. We found a correlation between the level of toxicity and proportion of CD4+ cells what may have an association with known presence of CD4+ lymphocyte at the site of skin toxic lesions (own observation, unpublished). In aGvHD CD4+ cells play a role at the level of alloantigen presentation but do not usually colonize the epithelium in which process CD8+ predominate [64]. 

In solid organ transplantation Treg cells mitigate chronic rejection but -not acute episodes [65,66]. It might be due to the complex relationship between CMV reactivation risk and Treg cells [67]. Pro-inflammatory cytokines likely generated early post-transplant increase the risk of CMV reactivation and this virus if activated has immunosuppressive potential e.g., encoding Il-10. Therefore CMV infection plays a role in both hematopoietic and solid organ transplantation shaping their outcome [68,69]. The complex relationship between the level of Tregs and alloreactive complications is also seen in patients post HSCT. It was shown that cGvHD but not aGvHD is characterized by high CD4+CD25 high lymphocytes values in blood [70,71,72]. Interestingly, higher values of CD4+CD25high lymphocytes were seen in the blood of patients at risk of *de novo* cGvHD (Figure 3) before the clinical symptomatology of this alloreactivity was clinically apparent This novel finding may assist the physician’s decision in planning the follow-up of patients post HSCT. This data helps to explain the lower incidence of aGvHD in patients who receive transplants from anti-CMV-IgG-positive donors. They have higher proportions of CD4+CD25high lymphocytes during the first wave of lymphocyte recovery. Interestingly, CD4+ lymphocytes were also higher in patients who received HSCT from anti-CMV-IgG-positive donors. However, in the multivariate analysis, only the level of CD4+CD25high lymphocytes played an independent and significant role in protection against aGvHD; no such effect was observed for the level of CD4+ lymphocytes. In our estimation, the higher values of CD4+ lymphocytes in the blood and a lower demand for IgG support of patients transplanted from anti-CMV-IgG positive donors reflect the higher potential of the immune system of patients constantly alerted by CMV repetitive reactivations. As a result at the early stages of chronic CMV infection the potential to combat infection may even increase [44,45].

In conclusion, the fate of HSCT patients depends significantly on the CMV status of the donors. If they are positive for anti-CMV-IgG antibodies, the immune system of recipients is doing better shortly after transplant, CD4+CD25high lymphocytes are in higher number what translates into the lower incidence of aGvHD. Higher levels of CD4+CD25high lymphocytes increase the chances of survival.
